# Assessing the construct validity of musculoskeletal ultrasound and the rheumatoid arthritis foot disease activity index (RADAI-F5) for managing rheumatoid foot disease

**DOI:** 10.1093/rap/rkad048

**Published:** 2023-05-11

**Authors:** Anika Hoque, Martijn Steultjens, Diane M Dickson, Gordon J Hendry

**Affiliations:** Department of Podiatry and Radiography, School of Health and Life Sciences, Glasgow Caledonian University, Glasgow, UK; Department of Podiatry and Radiography, School of Health and Life Sciences, Glasgow Caledonian University, Glasgow, UK; Department of Podiatry and Radiography, School of Health and Life Sciences, Glasgow Caledonian University, Glasgow, UK; Department of Podiatry and Radiography, School of Health and Life Sciences, Glasgow Caledonian University, Glasgow, UK

**Keywords:** RA, patient-reported outcome, RADAI-F5, musculoskeletal ultrasound, clinical examination

## Abstract

**Objective:**

The RA foot disease activity index (RADAI-F5) is a valid, reliable and clinically feasible patient-reported outcome measure (PROM) for the measurement of RA foot disease activity. Further validation of the RADAI-F5 against musculoskeletal ultrasonography (MSUS) for foot disease activity is necessary before clinical implementation. The aim of this study was to examine the construct validity of the RADAI-F5 in relationship to MSUS and clinical examination.

**Methods:**

Participants with RA completed the RADAI-F5. MSUS was used to evaluate disease activity (synovial hypertrophy/synovitis/tenosynovitis/bursitis) and joint damage (erosion) using greyscale (GS) and power Doppler (PD) at 16 regions in each foot, including joints and soft tissues. These same regions were examined clinically for swelling and tenderness. The construct validity of the RADAI-F5 was assessed using correlation coefficients and a priori*-*specified hypotheses for the strength of associations.

**Results:**

Of 60 participants, 48 were female, with a mean (s.d.) age of 62.6 (9.96) years and median disease duration of 15.49 (interquartile range 6–20.5) years. Theoretically consistent associations confirming construct validity [95% CI] were observed between the RADAI-F5 and MSUS GS (0.76 [0.57, 0.82]; strong), MSUS PD (0.55 [0.35, 0.71]; moderate), MSUS-detected erosions (0.41 [0.18, 0.61]; moderate), clinical tenderness (0.52 [0.31, 0.68]; moderate) and clinical swelling (0.36 [0.13, 0.55]; weak).

**Conclusion:**

Moderate to strong correlations between RADAI-F5 and MSUS demonstrate the good measurement properties of this instrument. With greater confidence in the utility of the RADAI-F5, clinical use of this new instrument as an adjunct to the disease activity score for 28 joints (DAS-28) could help to identify RA patients at risk for poor functional and radiological outcomes.

Key messages

- Moderate to strong associations between RADAI-F5 and musculoskeletal ultrasonography provide evidence of the good measurement properties of this instrument.
- RADAI-F5 associations with musculoskeletal ultrasonography-detected greyscale were stronger than clinical assessments of swelling and tenderness.
- The RADAI-F5 is a valuable tool for detecting foot disease activity in patients classified in DAS-28 remission.

## Introduction

RA is a chronic systemic inflammatory disease characterized by persistent synovitis with symmetric involvement of peripheral joints. RA often impacts peri-articular structures, such as synovial joints, bursae and tenosynovium [1]. Early diagnosis and an aggressive pharmacological treatment strategy have altered the prognosis for RA patients and placed more individuals into remission [2, 3]. Nevertheless, the prevalence of foot synovitis in RA patients remains high [4]. In 2010, the treat-to-target paradigm was proposed, with the therapeutic treatment objective of obtaining clinical remission or low disease activity [5]. This strategy relies on accurate monitoring of disease activity using composite DASs, including the clinical disease activity index (CDAI), the simplified disease activity index (SDAI) and most the most commonly used, DAS-28. Nonetheless, there are various criticisms of the DAS-28, including the subjective nature of clinical assessments of joint tenderness and swelling, the low specificity of the global visual analog scale [6] and its inability to detect foot arthritis [7]. Previous studies reveal that as many as one-third of patients classified in DAS-28 remission present with clinical and/or US-detected manifestations of foot synovitis, which might elevate their risk of radiographic joint degeneration and poor functional outcomes [5, 7]. In addition, although some patients might achieve clinical remission according to the DAS-28, they can still develop foot joint damage in the form of erosions, indicating that management driven by DAS-28 might not address foot disease adequately. Furthermore, a recent study indicated that clinical assessments for swollen and tender foot joints failed to identify the majority of individuals with self-reported foot pain [8]. This study suggests that foot joint counts should be complemented with other self-reporting measures for foot disease [8].

Prior studies have suggested the use of musculoskeletal ultrasonography (MSUS) for monitoring of foot disease in RA as a diagnostic aid to supplement clinical examination [8]. However, MSUS is not routinely used, largely owing to impracticalities, such as training needs and time to perform scans [8, 9]. For self-reporting, several RA foot-specific patient-reported outcome measures (PROMs) have been developed and validated to quantify foot impairments and disability in RA, such as the foot impact scale [10], foot function index [11] and Salford arthritis foot examination instrument [12]. Nonetheless, it appears that these PROMs lack clinical feasibility owing to their lengthiness and associated time burden for completion and scoring [13, 14]. Additionally, these PROMs focus on measurements of foot disability and impairment domains, which are less likely to have clinical utility for a treat-to-target approach with emphasis on early detection of disease activity and intervention to prevent poor outcomes. These limitations led to the development of the RA foot disease activity index (RADAI-F5), a valid, reliable, responsive and clinically feasible five-item PROM for quick and simple self-reporting of the presence and severity of foot disease in RA [13]. The RADAI-F5 was originally evaluated for its construct validity relative to global measures of disease activity, including the DAS-28 ESR and the modified RA disease activity index-5, and disease-specific measures for foot-related impairments and disability (the foot function index and foot impact scale) [13].

Qualitative research to identify barriers and facilitators to implementation of the RADAI-F5 in routine clinical practice suggested that the RADAI-F5 could be used to promote clinician–patient communication, to guide management, to screen patients and to monitor foot disease [14]. However, despite its apparent clinical utility, rheumatologists perceived the demonstration of construct validity of the RADAI-F5 relative to MSUS as crucial before widespread uptake and clinical implementation of this tool [14]. Accordingly, the aim of this study is to evaluate the construct validity of the RADAI-F5 compared with both MSUS and clinical examinations of foot joint and soft tissue tenderness and swelling.

## Methods

### Study design

From December 2021 to October 2022, this cross-sectional observational study involved a single visit to the MSUS imaging suite at Glasgow Caledonian University (GCU). Ethical approval was obtained from the North East Research Ethics Committee (21/NE/0130) and GCU Ethics School of Health Subcommittee **(**HLS/PSWAHS/20/242). Participants were recruited consecutively, and written informed consent was obtained from all participants. Our study conforms to the COnsensus-based Standards for the selection of health Measurement INstruments (COSMIN) Reporting Guideline for studies on measurement properties [15].

### Study participants

Adults with a diagnosis of RA in accordance with the 2010 revised ACR and EULAR criteria [16, 17] were recruited from National Health Service Greater Glasgow and Clyde rheumatology outpatient clinics at Gartnavel General Hospital, Royal Alexandra Hospital and University Hospital Wishaw. Patients were excluded if they were wheelchair users, had recent foot surgical interventions within the previous 12 months, had received foot/ankle CS injections within the previous 6 months or if they were diagnosed with co-morbidities that could have diminished their ability to distinguish between RA-related foot problems and problems attributable to other disease mechanisms, such as diabetes-related peripheral neuropathy. G Power determined that a sample size of 60 participants was needed for adequate detection of a correlation of ≥0.35 (weak), with a power of 80% and α-level set at 0.05.

### Data collection and measures

The participant assessments were all performed on the same day at the Glasgow Caledonian University Human performance laboratory. Demographic and clinical information was collected, including age, sex, disease duration, DAS-28 ESR scores and current medication [18, 19]. The modified version of the RA disease activity index (mRADAI-5) was collected as an additional self-reported measure of global disease activity [20]. Foot disease activity was evaluated using the RADAI-F5. The RADAI-F5 (Supplementary Data S1, available at *Rheumatology Advances in Practice* online) is a five-item questionnaire completed using a numerical rating scale format from 0 to 10 and scored by calculating an average summary score from the five items, ranging from 0 to 10 [13]. Foot disease remission state was defined as a RADAI-F5 score of ≤1.4, and foot disease categories for mild, moderate and high disease activity were defined as: >1.4 to ≤3.45, >3.45 to ≤5.7, and >5.7, respectively [13].

### Physical assessment

An independent assessor (G.J.H.) clinically examined 16 regions of each foot, including joints (ankle, subtalar, talonavicular, MTP joints two to five) and soft tissue sites (four intermetatarsal bursae, five plantar metatarsal bursae and the tibialis posterior tendon) for the presence of tenderness and swelling. Given that the questionnaires were locked in a secure locked safe until data collection was complete, G.J.H. completed the clinical evaluations blinded to the MSUS findings and questionnaire outcomes. Tender and swollen joint counts were each scored as present or absent, with a maximum score of 16 for tenderness and swelling for each foot (range 0–32 for both feet).

### Musculoskeletal ultrasonography

A single postgraduate certificate MSUS-trained podiatrist conducted all MSUS scans. MSUS assessment involved detection and grading of greyscale synovial hypertrophy (GS) and synovitis power Doppler (PD) signals of the same 16 regions of each foot, including joints (ankle, subtalar, talonavicular and MTP joints two to five) and soft tissue sites (four intermetatarsal bursae, five plantar metatarsal bursae and the tibialis posterior tendon). Longitudinal and transverse MSUS scans were performed with a Logiq S8 US machine (GE Medical Systems Ultrasound and Primary Care Diagnostics), using a linear transducer (9–15 MHz). Grading of each region was scored using a semi-quantitative scale of zero to three for GS and PD, as described in Table 1. Erosions and bursae were scored on a dichotomous scale as present or absent. The MSUS score was calculated as the summation of GS and PD scores (range 0–39) and erosions (range 0–7). The principal investigator (A.H.) completing the MSUS evaluation was blinded to the RADAI-F5 scores; however, it was not always possible to obtain an independent examiner to undertake the clinical examination owing to the coronavirus disease 2019 pandemic. As a result, whenever possible, A.H. was blinded to the clinical examination results (*n* = 44).

**Table 1.** Methods of scoring pathology on musculoskeletal ultrasonography

Pathology	Definition	Scoring
Synovial hypertrophy [21]	Abnormal hypoechoic, poorly compressible and non-displaceable intra-articular tissue, which may exhibit power Doppler signal	Score 0: no hypertrophy, independent of presence of effusion Score 1: minimal hypertrophy, with or without effusion up to level of horizontal line connecting bone surfaces Score 2: moderate hypertrophy, with or without effusion extending beyond joint line but with upper surface concave or hypertrophy extending beyond joint line but with upper surface flat Score 3: severe hypertrophy, with or without effusion extending beyond joint line but with upper surface convex
Synovitis [21]	Power Doppler interrogation of the synovial tissues	Score 0: no colour flow Score 1: ≤3 colour signals Score 2: <50% of the area filled with colour signals
Erosion [22]	Cortical break seen in longitudinal and transverse planes measuring >2 mm	Score 0: absent Score 1: present
Bursitis [23]	Hypoechoic, well-defined, anechoic or hypoechoic, compressible lesion, which may exhibit power Doppler signal	Score 0: absent Score 1: present
Tenosynovium hypertrophy [24]	Hypoechoic tendon thickening, with or without fluid in the tendon sheath, which may exhibit power Doppler signals, seen in two perpendicular planes	Score 0: normal (i.e. 6 mm in sagittal and 14.1 mm in transverse view) Score 1: minimal thickening of tendon Score 2: moderate thickening of tendon Score 3: severe thickening of tendon
Tenosynovitis [25]	Power Doppler interrogation of the tibialis posterior tendon in two planes	Score 0: no signals Score 1: signals in only one area of the tendon sheath Score 2: signals in more than one area of the widened tendon sheath Score 3: signals filling most of the widened tendon sheath

### Statistical analysis

All data were analysed using SPSS v.28 and Excel 2016. Descriptive statistics including age {median [interquartile range (IQR)]}, sex (female:male ratio) and disease duration (median [IQR]) were generated for all participants. The construct validity of the RADAI-F5 was assessed using correlation coefficients and a priori*-*specified hypotheses for the strength of associations. A priori hypotheses for construct validity included moderate positive correlations between RADAI-F5 scores and clinical examination, clinical tenderness, MSUS GS and PD, and a weak positive correlation with foot erosions. Pearson’s correlation and 95% CIs were used to test these hypotheses. Coefficients were interpreted as follows: 0–0.1 = negligible, 0.1–0.39 = weak, 0.4–0.69 = moderate, 0.7–0.89 = strong and 0.9–1.0 = very strong [26]. Participants were classified to corresponding foot and global disease groups based on RADAI-F5 and DAS-28 values (remission, low, moderate and high). To investigate MSUS-detected foot disease scores further, disease categories were cross-tabulated with MSUS scores for synovial hypertrophy (GS) and PD. This helped to determine the level of active disease in participants who met low disease and remission criteria based on DAS-28 and RADAI-F5 scores. To gain further insight into the behaviour of RADAI-F5 items, item-level associations with MSUS GS and PD scores were explored using Pearson’s correlations.

## Results

Sixty participants, of whom 80% were female, with a mean age of 62.4 (IQR 50–62) years and median disease duration of 120 months, participated in this study. Forty-three participants were receiving DMARD therapy, and 17 were receiving biologics. For DAS-28 ESR disease categories, 18% were in remission, 28% had low disease activity, 25% had moderate disease activity and 28% had high disease activity. For RADAI-F5 foot disease categories, 10% were in remission, 35% had low foot disease activity, 18% had moderate foot disease activity and 37% had high foot disease activity. Participants typically presented with moderate self-reported foot-related and global disease according to the RADAI-F5 and mRADAI-5 (Table 2). Tender joints and swollen foot joint and soft tissue sites were common, with a median (IQR) of two (four) and eight (one) observed, respectively. B-Mode GS was more prevalent than PD synovitis, with a mean (s.d.) of 14.85 (8.57) compared with 2.75 (3.23). Erosions were less frequently observed, with a mean (s.d.) of 0.70 (1.53).

**Table 2.** Participant descriptive data (*n* = 60)

Characteristic	All participants (*n* = 60)
Age, years	62.6 [9.97]
Sex, female:male	4:1
Disease duration, years	15.49 [12.19]
DAS-28 ESR	3.83 [1.38]
In remission (≤2.6), *n* (%)	11/60 (18)
Low disease (>2.6 to ≤ 3.2), *n* (%)	17/60 (28)
Moderate disease (>3.2 to ≤5.1), *n* (%)	15/60 (25)
High disease (>5.1), *n* (%)	17/60 (28)
Therapy	
DMARDs, *n* (%)	42/60 (70)
Biologic therapy, *n* (%)	17/60 (28)
Glucocorticoids between 6 months and 1 year, *n* (%)	12/60 (20)
None, *n* (%)	1/60 (2)
mRADAI-5	4.79 [2.05]
In remission (≤1.4), *n* (%)	3/60 (5)
Low disease (>1.6 to ≤ 3.0), *n* (%)	9/60 (15)
Moderate disease (>3.2 to ≤5.4), *n* (%)	23/60 (38)
High disease (>5.6), *n* (%)	25/60 (42)
RADAI-F5	4.39 [2.69]
In remission (≤1.4), *n* (%)	6/60 (10)
Low disease (>1.4 to ≤ 3.45), *n* (%)	21/60 (35)
Moderate disease (>3.45 to ≤5.7), *n* (%)	11/60 (18)
High disease (>5.7), *n* (%)	22/60 (37)
Clinical assessments	
TJC, median (IQR)	2 (1–3)
SJC, median (IQR)	8 (0–2)
GS	14.85 [8.57]
PD	2.75 [3.23]
Erosions	0.70 [1.53]

Results are shown as the mean [s.d.], unless specified.

DAS-28: DAS-28 joints; GS: greyscale; IQR: interquartile range; mRADAI-5: modified version of the RA disease activity index; PD: power Doppler; PROMIS-PF: patient-reported outcomes measurement information system—physical function short form questionnaire; RADAI-F5: RA foot disease activity index; SJC: swollen joint count; TJC: tender joint count.

Almost all of our participants’ self-reported the presence of foot or ankle disease 98% (59 of 60), of whom 32 of 60 (53%) were in the moderate/high RADAI-F5 categories. Foot synovial hypertrophy, defined as having a GS score of at least two, was prevalent in 57 of 60 (95%) participants. Foot synovitis, defined as having a PD score of at least one, was evident in 38 of 60 (63%) participants. Synovial hypertrophy observed by MSUS was most prevalent in the second and third MTP joints and ankle joint, whereas the tibialis posterior was the most affected soft tissue site. MSUS-detected synovitis was more frequently observed at the ankle joint. The most common sites for clinical detection of swelling were the ankle and subtalar joints, whereas the most common sites for clinical detection of tenderness were the intermetatarsal bursae, the ankle joint and the second and third MTP joints.

### Associations between RADAI-F5 and MSUS and clinical examinations of foot disease

**Figure 1.**
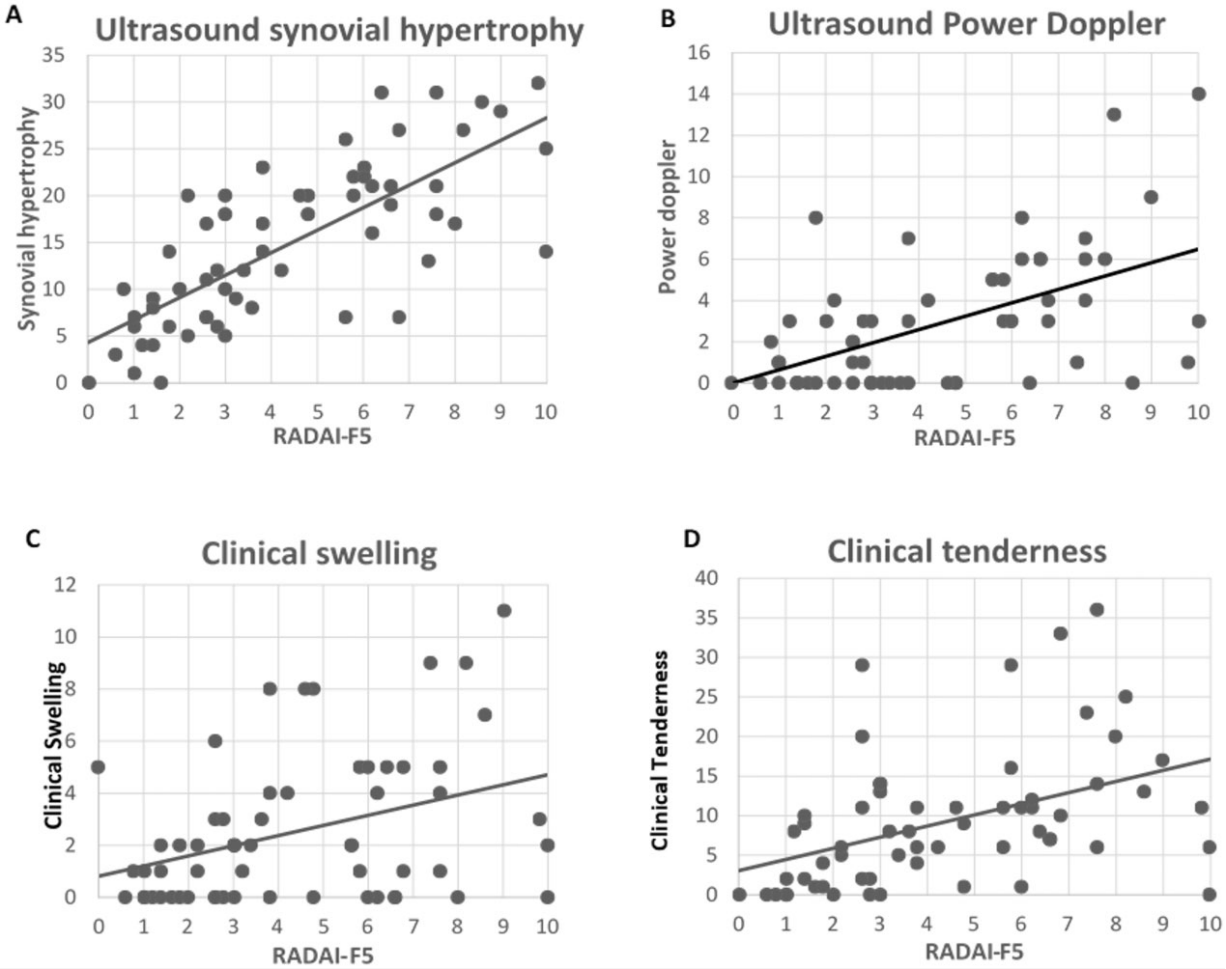
Associations of the RA foot disease activity index with synovial hypertrophy (**A**), power Doppler (**B**), clinical swelling (**C**) and clinical tenderness (**D**) RADAI-F5: RA foot disease activity index

The association between the RADAI-F5 and GS synovial hypertrophy was stronger than expected (Pearsons (*r)* = 0.75 [95% CI 0.61, 0.84], *P* < 0.01; Table 3; Fig. 1). As anticipated, a moderate positive association was observed with PD (*r* = 0.60 [95% CI 0.41, 0.74], *P* < 0.01) and a weak association with erosions (*r* = 0.29 [95% CI 0.04, 0.51], *P* < 0.01). The RADAI-F5 had a weaker than anticipated association with clinical swelling (*r* = 0.37 [95% CI 0.13, 0.57], *P* < 0.05) and a moderate association with clinical tenderness (*r* = 0.44 [95% CI 0.21, 0.62], *P* < 0.01; Fig. 1). Construct validity was confirmed, with 60% of associations in line with the a priori hypotheses.

**Table 3.** Pearson’s correlations of RA foot disease activity index with objective measures of foot disease activity for a priori hypothesis testing

Measure (*n* = 60)	Correlation coefficient (95% CI)	Strength	*A priori* hypothesis
Clinical swelling	0.37 (0.13, 0.57)	Weak	Moderate
Clinical tenderness	0.44 (0.21, 0.62)	Moderate	Moderate
Synovial hypertrophy (GS)	0.75 (0.61, 0.84)	Strong	Moderate
Synovitis (PD)	0.60 (0.41, 0.74)	Moderate	Moderate
Erosions	0.29 (0.04, 0.51)	Weak	Weak

GS: greyscale; PD: power Doppler.

### Comparison of RADAI-F5, DAS-28 and MSUS characteristics of the low foot disease/remission foot groups

Seventeen individuals with RA scored positively on the RADAI-F5 despite not having active foot disease as indicated by MSUS. Compared with the moderate–severe RADAI-F5 group, the remission and low disease activity RADAI-F5 group scored significantly higher for item 1 (related to foot disease activity in the previous 6 months), with a mean (s.d.) item score of 2.11 (1.57) and 4.57 (2.28), respectively. Calculating the correlation coefficient for each RADAI-F5 item against MSUS GS and PD findings revealed that the association between RADAI-F5 items and PD exhibited weak positive associations for questions related to foot disease in the last 6 months (item 1) and morning stiffness (item 5). In comparison, GS scores were moderately associated only for questions related to morning stiffness (item 5). Items related to joint tenderness/swelling (item 2), pain (item 3) and foot health (item 4) were strongly associated with GS and moderately associated with PD (Table 4).

**Table 4.** RA foot disease activity index item associations with musculoskeletal ultrasonography

RADAI-F5 Items	GS MSUS [correlation coefficient (95% CI)]^a^	Strength	PD MSUS [correlation coefficient (95% CI)]^a^	Strength
Item 1	0.62 (0.44, 0.76)	Moderate	0.45 (0.22, 0.63)	Moderate
Item 2	0.75 (0.61, 0.84)	Strong	0.57 (0.37, 0.71)	Moderate
Item 3	0.73 (0.59, 0.83)	Strong	0.58 (0.38, 0.73)	Moderate
Item 4	0.68 (0.52, 0.80)	Moderate	0.62 (0.44, 0.76)	Moderate
Item 5	0.59 (0.40, 0.73)	Moderate	0.44 (0.21, 0.62)	Moderate

a Pearson’s correlations, all significant at *P* < 0.05.

GS: greyscale; MSUS: musculoskeletal ultrasonography; PD: power Doppler.

Patients in DAS-28 remission scored mean (s.d.) RADAI-F5 scores of 2.67 (2.13). Of the 11 patients classified according to DAS-28 remission, 4 patients remained in remission according to the RADAI-F5, 5 patients were in RADAI-F5 low disease category, and 2 were classified as having high levels of foot disease. The analysis of the DAS-28 categories against MSUS features revealed that 63% of individuals in the DAS-28 remission category had at least grade 2 GS at one or more sites of interest (as seen in Table 5). In comparison, 88% of individuals in the DAS-28 low disease category had at least grade 2 GS, while 100% of those in moderate to high DAS-28 categories had at least grade 2 GS. Furthermore, 54% of those in the low DAS-28 category and 53% of those in the remission DAS-28 category still had PD signals in the foot structures of interest (as seen in Table 5).

**Table 5.** DAS-28 disease category summary statistics

DAS-28	GS MSUS ≥ grade 2 [*n* (% affected)]	PD MSUS ≥ grade 1 [*n* (% affected)]	MSUS erosion at more than one site [*n* (% affected)]
In remission (*n* = 11)	7 (64)	6 (54)	0 (0)
Low (*n* = 17)	15 (88)	9 (53)	3 (18)
Moderate (*n* = 15)	15 (100)	9 (60)	6 (40)
High (*n* = 17)	17 (100)	13 (77)	11 (65)

GS: greyscale; MSUS: musculoskeletal ultrasonography; PD: power Doppler.

## Discussion

To our knowledge, this is the first study to use the RADAI-F5 to examine the relationship between MSUS-detected and self-reported foot disease activity. The RADAI-F5 demonstrates good construct validity, in line with a priori expectations, suggesting that this new tool has moderate to strong associations with MSUS GS, PD and erosions. This work provides additional evidence to our previous validation work [13]. The DAS-28 classed *n* = 11 participants in remission; of these, 54% presented with at least grade 1 PD affecting at least one site of interest. In addition, *n* = 17 individuals were assigned to the DAS-28 low disease category, and 53% presented with at least grade 1 PD affecting at least one site of interest within the foot. Nevertheless, this result was anticipated based on evidence that composite disease activity indices that exclude foot joints might not represent foot synovitis accurately [13]. Consequently, systemic therapy escalated solely on the basis of DAS-28 values and in the context of infrequent foot examinations during routine consultations might result in persistent foot disease and suboptimal management. Our findings demonstrate that the RADAI-F5 has clinical utility in detecting the activity of inflammatory foot disease in individuals with established and early RA, and that monitoring the feet is crucial for these patients owing to the high incidence of foot involvement in RA. The incidence of foot involvement in RA has been described extensively in the literature, with earlier estimates ranging from 56 to 100% [13, 27]. Despite advancements in pharmaceutical management, the prevalence of foot disease remains high in our sample, as evidenced by the 95% of participants who exhibited MSUS-detected GS in the foot and 64.4% who had active PD signals in the foot or ankle.

In clinical practice, people with RA are routinely evaluated by the number of swollen and tender joints to determine disease activity. The addition of a 12-joint foot count to the DAS-28 in a foot or ankle pain detection study showed that 92.3% and 61.4% of patients with foot pain had no swollen or tender foot joints, respectively, revealing a significant discrepancy between clinical examination and self-reported foot pain [13]. The authors speculated that the high incidence of foot pain might be attributable to structures not included in the 12-joint foot count, such as the midfoot and other soft tissue structures. Despite this, the vast majority of our patients did not test positive for oedema, even when soft tissue structures and hindfoot joints were included in the assessment. This suggests that clinical examination undertaken in isolation, without additional MSUS imaging and/or a valid method of foot disease self-report, might be a suboptimal approach. Incorporating the RADAI-F5 as an adjunct to the clinical examination might assist in the identification of more individuals with active foot disease.

Several individuals (*n* = 17) scored higher than zero on the RADAI-F5 despite the absence of synovitis, tenosynovitis or erosions in their foot and ankle joints. Those in remission and low RADAI-F5 disease categories scored the highest on item 1 (related to foot disease activity in the previous 6 months), indicating that there might have been active disease in the past that was not present on the day of the MSUS scan and residual foot symptoms attributed to previous active foot disease. Despite the absence of active Doppler signals, the participants in the high RADAI-F5 disease group appeared to have GS scores typically grade 2 or 3, indicating that the presence of synovial hypertrophy is strongly associated with self-perceptions of foot disease. Typically, the primary focus of pharmacological treatment for RA is the inflammatory component of the disease, which is identified by Doppler activity rather than synovial hypertrophy [28, 29]. Witt *et al.* [30] found that grade 1 synovial hypertrophy is evident in healthy people and is unresponsive to therapy in early and established RA, and Padovano *et al.* [31] identified similar findings in several healthy controls with grade 1 synovial hypertrophy. Nonetheless, Terslev *et al.* [32] reported that grade 1 synovial hypertrophy could improve with the initiation of biological treatment, regardless of the absence of Doppler activity. Similar outcomes have been observed for tenosynovitis, as grade 1 tenosynovitis without positive Doppler activity improves with therapy [33]. Moreover, synovial hypertrophy in RA patients has been associated with an early recurrence of inflammatory arthritis and is predictive of erosion progression [34, 35]. This indicates that GS synovial hypertrophy is still meaningful and responsive to change and that eradication of Doppler signals might not be the main therapeutic aim in RA patients when contemplating therapy escalation. In accordance with OMERACT [21], synovial hypertrophy without Doppler activity is a symptom of active disease and should be considered when assessing the activity of foot disease using MSUS. As such, our findings might indicate that synovial hypertrophy still impacts upon self-perceptions of foot disease. Strong associations between GS and RADAI-F5 are therefore encouraging, because they suggest that individuals with RA might be able to detect localized inflammatory alterations in synovial hypertrophy and indicate the possible use of RADAI-F5 as a screening tool.

It is noteworthy that the RADAI-F5 does not specify which areas of the foot are affected; rather, it offers a comprehensive score for foot disease. Use of the RADAI-F5 in podiatry and rheumatology clinics has the potential to enhance the patient experience and quality of care by facilitating early detection of RA-related foot disease and informing therapeutic approaches based on RADAI-F5 disease classification categories [13]. We suggest that patients with RA be offered the opportunity to complete the RADAI-F5 in the waiting area before their appointment. We recommend providing patients in RADAI-F5 remission and with low disease with verbal and written information regarding their condition and its management, in addition to footwear guidance and, if needed, functional orthotics. A RADAI-F5 score in the moderate and high categories should prompt additional inquiry by clinical examination of foot joints and soft tissues, or patients can complete a foot map, in which they shade problematic areas. If clinical examination verifies the existence of suspicious joints, we suggest considering MSUS imaging to confirm the presence of synovitis, in which case CS injections would be recommended. In addition, allied health-care providers should consider referral to rheumatology for possible medication escalation. The RADAI-F5 can also be used in remote consultations to identify patients who necessitate immediate in-person appointments, where clinical or MSUS assessments are advisable.

There are some limitations to this study. Firstly, quantifying the symptomatic impact of subclinical foot synovitis is difficult; thus, longitudinal follow-up is required. Specifically, longitudinal data following pharmacological treatment are necessary to evaluate the relative responsiveness of MSUS to establish whether the RADAI-F5 can evaluate the effectiveness of therapy in clinical care. Secondly, there is a risk of selection bias because individuals who participated in the research were recruited from a rheumatology outpatient clinic, who might have experienced a more significant degree of foot involvement than patients in DAS-28 remission on or off medication, who would be less likely to attend as frequently. Another limitation of this study is that the MSUS examiner was not blinded to all clinical foot examinations, thus investigator bias cannot be discounted. Nonetheless, given that the RADAI-F5 scores were concealed from the lead investigator, it is unlikely that the association between RADAI-F5 scores and MSUS-detected foot disease was compromised.

The present study found that RA foot and ankle disease remains prevalent even in patients in DAS-28 remission. As anticipated, the correlation between RADAI-F5 scores and MSUS-detected synovial hypertrophy was more significant than clinical assessments of joint swelling and tenderness. With greater confidence in the utility of the RADAI-F5, clinical use of this new instrument could help to identify RA patients at risk for poor functional and radiological outcomes. Adopting the RADAI-F5 as an adjunct to composite disease activity indices, such as the DAS-28, might improve local disease detection and guide new foot care protocols and has potential for use in a wide range of clinical applications, including triage, improved communication with patients and multidisciplinary teams, and remote consultations.

## Supplementary Material

rkad048_Supplementary_Data

## Data Availability

The data underlying this article cannot be shared publicly due to the privacy of individuals that participated in the study. The data will be shared on reasonable request to the corresponding author.
