# Supplementary figures and images for: Assessing the construct validity of musculoskeletal ultrasound and the rheumatoid arthritis foot disease activity index (RADAI-F5) for managing rheumatoid foot disease
Source: Rheumatol Adv Pract. 2023 May 11;7(2):rkad048. doi: 10.1093/rap/rkad048 (PMC10224803; doi:10.1093/rap/rkad048)

# **Supplementary Data S1: RADAI-F5**


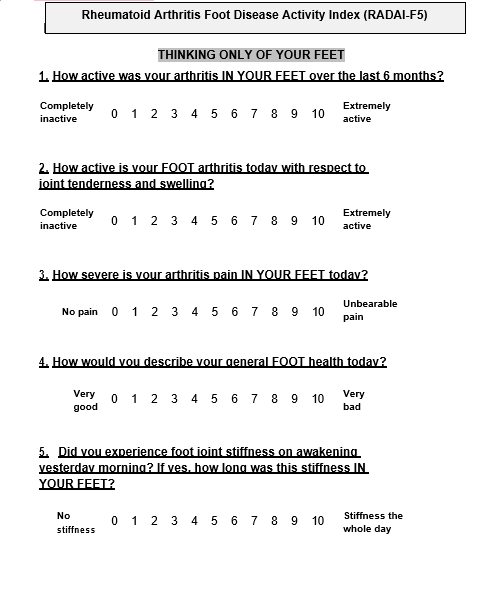

Supplement: rkad048_Supplementary_Data [file rkad048_supplementary_data.docx]
